# Complex chromosome rearrangements related 15q14 microdeletion plays a relevant role in phenotype expression and delineates a novel recurrent syndrome

**DOI:** 10.1186/1750-1172-6-17

**Published:** 2011-04-19

**Authors:** Maria Cristina Roberti, Cecilia Surace, Maria Cristina Digilio, Gemma D'Elia, Pietro Sirleto, Rossella Capolino, Antonietta Lombardo, Anna Cristina Tomaiuolo, Stefano Petrocchi, Adriano Angioni

**Affiliations:** 1 Cytogenetics and Molecular Genetics Unit - Bambino Gesù Children's Hospital, Rome 00165, Italy; 2 Medical Genetics Unit - Bambino Gesù Children's Hospital, Rome 00165, Italy

## Abstract

Complex chromosome rearrangements are constitutional structural rearrangements involving three or more chromosomes or having more than two breakpoints. These are rarely seen in the general population but their frequency should be much higher due to balanced states with no phenotypic presentation. These abnormalities preferentially occur *de novo *during spermatogenesis and are transmitted in families through oogenesis.

Here, we report a *de novo *complex chromosome rearrangement that interests eight chromosomes in eighteen-year-old boy with an abnormal phenotype consisting in moderate developmental delay, cleft palate, and facial dysmorphisms.

Standard G-banding revealed four apparently balanced traslocations involving the chromosomes 1;13, 3;19, 9;15 and 14;18 that appeared to be reciprocal. Array-based comparative genomic hybridization analysis showed no imbalances at all the breakpoints observed except for an interstitial microdeletion on chromosome 15. This deletion is 1.6 Mb in size and is located at chromosome band 15q14, distal to the Prader-Willi/Angelman region. Comparing the features of our patient with published reports of patients with 15q14 deletion this finding corresponds to the smallest genomic region of overlap. The deleted segment at 15q14 was investigated for gene content.

## Background

Chromosomal abnormalities are the most commonly recognized causes of developmental delay and mental retardation, accounting for approximately 10% of cases [1].

High-resolution molecular methods, i.e. array-based comparative genomic hybridization (array-CGH) allow a careful characterization of unbalanced rearrangements, enabling a more explicit genotype/phenotype correlation [2] and enhancing the capacity to map disease-causing genes [3].

Complex chromosome rearrangements (CCRs) are defined as constitutional structural chromosomal rearrangements with at least three cytogenetically visible breakpoints and exchange of genetic material between two or more chromosomes [4]. These are rare, although clinically important to recognize, because carriers can have phenotypes spanning from normal individuals, infertile males, mental retardation, to congenital abnormalities and they can be responsible for recurrent miscarriages in females [5-7].

The alterations can arise *de novo *or be familial; familial CCRs tend to involve less chromosomes and fewer breakpoints than *de novo *CCRs [8]. A survey of 269371 prenatal studies reported a total of 246 apparently cytogenetically balanced anomalies; among them, 3% were *de novo *presumably balanced CCRs [7]. There is a high prevalence of maternal origin in familial CCRs and a high incidence of mental retardation and phenotypic abnormalities in *de novo *CCRs, but in rare occasions they can be found in phenotypically normal individuals [9]. These rearrangements preferentially occur *de novo *during spermatogenesis and are transmitted in families through oogenesis.

In *de novo *CCRs, associated with mental retardation, the degree of severity correlates with the number of breakpoints [5,8,10,11]. According to the number of chromosomes breaks, CCRs are classified as type I (3 or 4 breaks) and type II (5 or more breaks) [10,12].

In the past, size and banding pattern of the interested segments as well as the number of chromosomes involved could hamper delineation of the correct karyotype. Moreover, the conventional cytogenetics was of limited use in determining whether a CCR was balanced or unbalanced. Despite the importance of refining the multiple rearrangement breakpoints at the sequence level in CCR cases, virtually no breakpoints have been sequenced and no molecular mechanisms have been proposed for how they might occur. To date, most of the breakpoints have been mapped using conventional cytogenetic G-banded karyotyping, multi-subtelomeric fluorescence in situ hybridization (FISH), whole chromosome painting FISH, multicolor FISH (M-FISH) or spectral karyotyping (SKY) or multicolor banding (MCB) [5,6,13].

More recent studies have used array-CGH to uncover cryptic rearrangements [13]. Deletions at the breakpoint regions are a common finding, but duplications are also detected [6,14]. Importantly, when the resolution of the analysis methods used to examine CCRs improves, the initially identified number of breakpoints tends to increase [14-16]. This observation suggests that many, and possibly the majority of CCRs detected to date might actually be more complex than initially thought. In fact, De Gregori et al. [14] reported that 40% of patients observed as 'balanced translocations' were unbalanced and, remarkably, 18% of the reciprocal translocations were, instead, complex rearrangements.

After reviewing 226 CCRs reported in the literature, it is possible to observe a clear chromosome preference in CCRs events. In fact, the most common chromosomes involved in CCRs reported in the literature are 2, 3, 4, 7, 11 with frequencies of approximately 10-12% [13].

Here, we describe a *de novo *complex chromosome rearrangement, involving eight chromosomes, with a submicroscopic deletion in 15q14 in a boy with moderate mental retardation, cleft palate and facial anomalies. We discuss the implications of this deletion for identifying candidate genes related to the clinical features.

## Case Presentation

### Patient's description

This boy is the second child of healthy, nonconsanguineous Caucasian parents. At birth the mother was 31 years old, the father 29. Family history showed mental retardation in the sister of the proband's maternal grandfather. The patient was born by Cesarean section at term of an uneventful pregnancy. Birth weight was 3100 g (25^th ^centile), length 49 cm (25^th ^centile), head circumference 34 cm (25^th ^centile). Apgar scores were 7 and 8 at 1 and 5 minutes, respectively. Cleft palate was diagnosed at birth, and repaired at 8 months of age. Developmental milestones were retarded (sitting at 12 months, walking at 30 months). Language was delayed. Learning difficulties were recorded and the patient needed special assistance at school.

**Figure 1:**
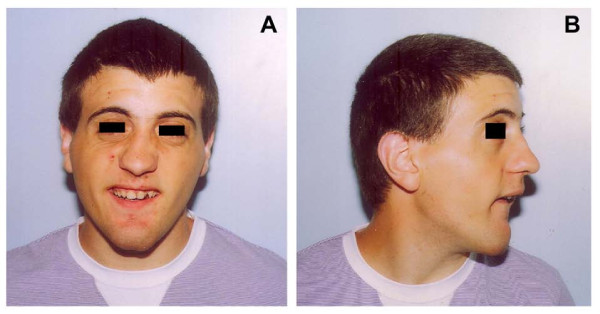
**Facial appearance of the patient**. Front view (A) and side view (B).

The patient was first evaluated by us at the age of 18 years and 6 months. Weight was 56.5 kg (10^th^-25^th ^centile), height 154 cm (<3^rd ^centile), head circumference 53.5 cm (3^rd ^centile). Facial anomalies included bitemporal narrowing, deep-set eyes, short and smooth philtrum, squared pointed chin, irregular dentition, multiple acne lesions (Figure 1 A and 1B). Short stature was proportionate.

The boy was evaluated only by cerebral CT scan and it showed hypoplasic frontal lobes. The patient's parents refused cerebral MRI. Electroencephalogram, color-Doppler echocardiography and renal ultrasonography were normal. Ophthalmological and audiological examinations revealed no anomalies. Bone age at 8 years was corresponding to chronological age. Vertebral column X-ray showed mild kyphoscoliosis.

### Classical and molecular cytogenetic studies

Phytohemagglutinin stimulated peripheral blood lymphocytes from the patient and his parents were short term cultured and the metaphases obtained were karyotyped with GTG banding. The karyotypes were described according to the International System for Human Cytogenetic Nomenclature (ISCN, 2005) [17].

To better recognize the chromosome segments involved in the rearrangements a panel of commercially available probes was used in Fluorescence In Situ Hybridization (FISH) experiments. DNA probes selected were: Prader-Willi Syndrome (PWS) on 15q11-13 (Vysis); Retinoblastoma (RB1) on 13q14 (Vysis); whole painting probes specific for chromosomes 1, 3, 9, 13, 14, 15, 18, 19 (Metasystems, Altlussheim, Germany); α-satellite probes for centromeres of chromosomes 13/21 (Q-BIOgene, Illkirch, France).

For breakpoints characterization of the complex chromosome rearrangement, bacterial artificial chromosome (BAC) clones were used as probes in FISH analysis. They were selected according to the UCSC database University of California Santa Cruz, [http://genome.ucsc.edu/], March 2006 release, and are listed in Table 1. The BACs belonging to the Roswell Park Cancer Institute library [http://www.chori.org/bacpac/] were obtained from Resources for Molecular Cytogenetics [http://www.biologia.uniba.it/rmc/].

**Table 1:** List of the probes used in FISH experiments

TRASLOCATION	PROBE	CHROMOSOME BAND	RESULT
(1;13)	wcp 1	# 1	1, der(13)
	RP11-433N2	1p31.2	1, der(13)
	RP11-149B17	13q12.11	13, der(13)
	RB1	13q14.2	13, der(1)

(3;19)	wcp 3	# 3	3, der(19)
	wcp 19	# 19	19,der(3)

(9;15)	wcp 9	# 9	9,der(15)
	RP11-59O6	9p24.3	9, der(15)
	α satellite	15p11.1-q11.1	15, der(15)
	*SNRPN*	15q11-13	15, der(15)
	*PML*	15q24.1	15, der(9)

(14;18)	wcp 14	# 14	14, der(18)
	RP11-151D11	18p11.21	18, der(18)
	RP11-138C24	18p11.31	18, der(14)

Clones utilized to further investigate the breakpoints of the complex chromosome rearrangement in our patient.

FISH experiments were carried out as previously described [18]. The probes were directly labeled with Cy3-dUTP or fluorescein-dUTP (Perkin Elmer Life Sciences, Boston, MA, USA). Digital images were obtained using a Nikon Eclipse E1000 epifluorescence microscope equipped with a cooled CCD Photometrics CoolSNAP FX camera. Pseudocoloring and merging of images were performed with Genikon software v3.6.16.

### Molecular analysis

DNA was extracted from peripheral blood of the patient and his parents with High Pure PCR Template Preparation Kit (Roche, Mannheim, Germany) according to the producer's instructions.

Molecular experiments were performed in order to assess the parental origin of the chromosome 15q14 deletion with a panel of short tandem repeats (STRs) using multiple primer pairs (available upon request) obtained from UniSTS database included in NCBI [http://www.ncbi.nlm.nih.gov/unists/]. DNA was amplified following standard protocol by means of GeneAmp PCR System 2700 (Applied Biosystems, Foster City, CA, USA) following standard protocol. One primer from each pair was fluorescently labelled and PCR products were run on an ABI Prism 310 (Applied Biosystems), using GeneMapper v 3.0 as software.

### Array-CGH

The array-CGH (comparative genomic hybridization) studies of the patient and his parents were performed using Agilent Technologies Array-CGH Kits (Santa Clara, CA, USA). This platform is a 60-mer oligonucleotide-based microarray that allows molecular profiling of genomic aberrations with an overall median probe spatial resolution of 20 kb (105K) and a probe spacing in RefSeq Genes of 18.9 kb (105K).

Aliquots of 2000 ng of DNA from patient, parents and same-sex reference home made pool were double-digested with *RsaI *and *AluI *for 2 hours at 37°C. After heat inactivation of the enzymes at 65°C for 20 minutes, each digested sample was labelled by random priming (Agilent Technologies) for 2 hours using Cy5-dUTP for patient/parent DNAs and Cy3-dUTP for reference DNAs. Labelled products were column purified with Illustra CyScribe GFX purification kit (GE Healthcare, Buckinghamshire, UK). After probe denaturation and pre-annealing with 5-25 mg of Cot-1 DNA, hybridization was performed at 65°C with rotation for 40 hours (105K). After washing steps, following the manufacturer's instructions, the array was analyzed using an Agilent scanner and Feature Extraction software v.10.5. A graphical overview of the results was obtained using DNA Analytics software v.4.0. The chromosome aberration regions were calculated by ADM1 algorithm with moving average window of 1Mb.

In order to confirm the results obtained FISH experiment was performed using the BAC clones RP11-698F12 (accession number AC087487, chr15:33,659,677-33,839,903 bp) and RP11-203K2 (chr15:34,350,630-34,527,067 bp).

### Bioinformatic analysis

In order to evaluate if the Copy Number Variations (CNVs), detected by array-CGH, were polymorphic or potentially correlated with the clinical phenotype of our patient, bioinformatic analysis was carried out consulting the Database of Genomic Variants BioXRT [http://projects.tcag.ca/variation/].

With the aim to disclose the mechanisms underlying the chromosome interstitial microdeletion and to estimate if the genes included were imprinted, the Human Genome Segmental Duplication Database [http://projects.tcag.ca/humandup/] and the Gene Imprint Database [http://www.geneimprint.com/] were queried.

Investigation of gene contents in the deleted segment was carried out comparing the "UCSC Genes based on RefSeq, UniProt, GenBank, CCDS and Comparative Genomics" track (March 2006 release, hg18) with the corresponding interval in the "RefSeq Genes" track in the UCSC last release (Feb 2009, hg19).

## Results

**Figure 2:**
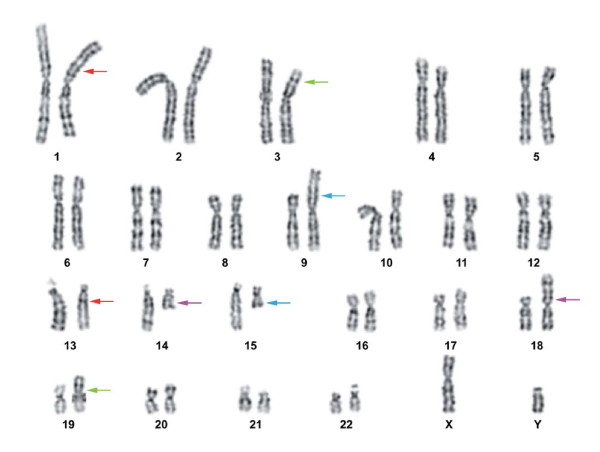
**Cytogenetic analysis**. Karyotype from a peripheral blood metaphase of the patient: 46,XY,t(1;13)(p31.1;q13),t(3;19)(p23;p12),t(9;15)(p23;q14),t(14;18)(q22;p11.23). The arrows of the same color indicate the breakpoints of a reciprocal translocation.

The G-band analysis at 550 band resolution revealed a complex karyotype with four independent, apparently balanced, reciprocal traslocations in all the metaphases analyzed. The karyotype was as follows (Figure 2): 46,XY,t(1;13)(p31.1;q13),t(3;19)(p23;p12),t(9;15)(p23;q14),t(14;18)(q22;p11.23). Parental G-banded karyotypes were normal.

**Figure 3:**
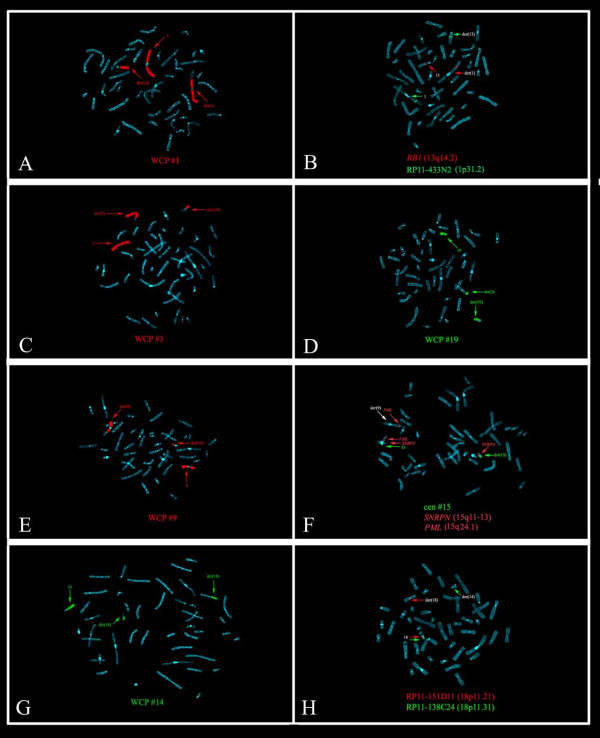
**FISH results**. Two pictures for each reciprocal traslocation of the complex chromosome rearrangement are showed: t(1;13) (A) painting #1 and (B) co-hybridization between the BAC probe RP11-433N2 green (1p31.2) with the probe RB1 red (13q14.2) specific for the retinoblastoma gene; t(3;19) (C) painting #3 and (D) painting #19; t(9;15) (E) painting #9 and (F) probe PWS specific for the Prader-Willi critical region (*SNRPN*) red (15q11-13), centromeric probe for the chromosome 15 in green and *PML *in red (15q24.1) as controls; t(14;18) (G) painting #14 and (H) dual color FISH experiment with BACs RP11-151D11 red (18p11p.21) and RP11-138C24 green (18p11.31).

Results of the FISH experiments, carried out to further characterize the complex chromosome rearrangement, were reported in Table 1 and Figure 3.

**Figure 4:**
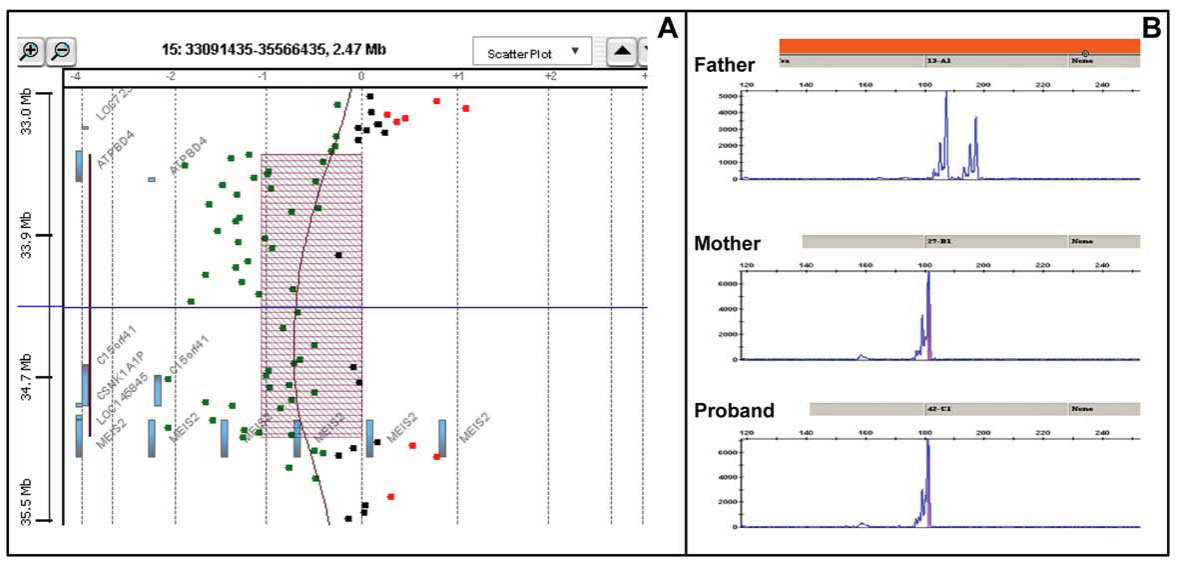
**Molecular analysis**. Array-CGH profile of the 1.6 Mb deleted region at 15q14 (A). Microsatellite analysis performed using informative STRs (D15S1042 and D15S118) included in the deleted region, showed allelic loss of heterozygosity and revealed the paternal origin of the rearrangement (B).

To search for possible cryptic imbalances at the breakpoints, array-CGH study was then performed with Agilent 105K Array (Agilent Technologies), revealing a submicroscopic heterozygous interstitial deletion on chromosome 15q14 extending from 33,471,941 to 35,072,476 bp positions (hg 18, NCBI Build 36.1, March 2006) and containing 51 oligonucleotides (Figure 4A). The rearrangement was confirmed by FISH with the BAC clones RP11-698F12 and RP11-203K2 that resulted deleted on the derivative chromosome 15.

Moreover, the deleted region was analyzed for the presence of segmental duplication and potentially imprinted genes but they were not found.

Additional information was obtained from microsatellite analysis carried out to assess the parental origin of the defect. The informative STRs (D15S1042 and D15S118) included in the deleted region, showed allelic loss of heterozygosity, revealing that this complex rearrangement arose in the paternal meiosis (Figure 4B).

## Discussion and Conclusions

Abnormal phenotypes observed in persons who harbor apparently balanced chromosomal rearrangements are thought to result from disruption of gene(s) at chromosome breakpoint(s), from undetected additional genomic imbalance by routine karyotyping or from position effect [19]. Recently, high resolution genome wide array-based analyses have enabled identification of previously unknown submicroscopic abnormalities at the traslocation breakpoints or in other genomic regions in patients with CCRs [6,14,20-22].

In the present paper, we reported a *de novo *complex cytogenetic profile interesting eight chromosomes. G-banding analysis showed eight chromosomes (1, 3, 9, 13, 14, 15, 18 and 19) involved in the CCR with four reciprocal traslocations and eight breakpoints. According to the number of chromosome breaks, our case would be classified as type II (5 or more breaks) [10,23]. Zhang et al [13] reviewed the frequency of specific chromosomes in 226 CCR cases reported in the literature. Comparing the rearranged chromosomes of our patient with those listed by Zhang, they are rarely involved in CCRs, except for the chromosome 3 that shows a frequency of approximately 10%. The reason for this preference is not obvious.

Array-CGH studies were needed to obtain a more precise delineation of the structural abnormalities and they revealed a cryptic microdeletion at 15q14, 1.6 Mb in size, overlapping with the breakpoint previously identified by means of G-banding analysis. The parental origin of this deletion was found to be paternal, the CCR occurring during spermatogenesis. Interestingly, the origin of *de novo *CCR and reciprocal translocation cases are frequently reported as paternal [8,14,21,23,24]. The cause of CCRs is unknown, however, the fact that all reported cases have been non-mosaic and have involved only one chromosome of a homologous pair suggests a 'catastrophic' meiotic event in one of the parental gametes rather than a post-zygotic event. One considered aspect is the influence of the environment on the genome. In fact, some authors [23] have observed a correlation between the exposure to radiations and CCRs. In our case, no particular risks for radiation damage had been reported by the parents.

The deleted segment at 15q14 was investigated for gene content. Three known genes (*ATPBD4*, ATP binding domain 4; *C15orf41*, 15 open reading frame 41; *MEIS2*, Meis homeobox 2), one pseudogene (*CSNK1A1P*, casein kinase 1, alpha 1 pseudogene) and one predicted gene (*LOC145845*) were found.

The protein encoded by *ATPBD4*, located on the minus strand, has a domain conserved from chimpanzee to yeast forming an alpha/beta/alpha fold which binds to adenosine nucleotide.

*C15orf41 *encodes the predicted protein LOC84529, with two isoforms, one 281 and two 183 aminoacids in size, whose functions still remain unclear.

*MEIS2 *encodes a homeobox protein belonging to the TALE ('three amino acid loop extension') family of homeodomain-containing proteins. TALE homeobox proteins are highly conserved transcription regulators, and several members have been shown to be essential contributors to developmental programs. Multiple transcript variants encoding distinct isoforms have been described for this gene and the longest one contains 12 exons. The gene is transcribed on minus strand and is about 200 kb in size. The breakpoint in our case is located in a short segment of about 20 kb between the last deleted probe (A_16_P2021458; 35,072,417-35,072,476) and the first conserved probe (A_14_P124780; 35,094,819-35,094,878) producing the deletion of about half the gene with the loss of 4 exons mapping at the 3' end. Although the deletion is about 100 kb, the first 8 exons were conserved.

The deletion of *MEIS2 *has been recently reported in patients with cleft palate and congenital heart defects [3] suggesting its involvement also in the clinical phenotype of our patient. Moreover, Stankunas et al [25] showed that disruptions of *MEIS1*, a gene belonging to the same family of *MEIS2 *and interacting with *PBX1-2-3 *through the formation of a heteroligomeric complexes, produces heart defects in mice because it controls a subset of target genes that regulate cardiac outflow tract formation.

We have compared the clinical features of our patient with published reports of patients with larger, cytogenetically visible deletions encompassing chromosome band 15q14, as listed in Table 2[3,26-38]. These comparisons are, however, hampered by additional chromosome aberrations present in about half (7/15) of these patients, and the different deletion sizes.

**Table 2:** Clinical features of the patients with deletions including cytogenetic band 15q1 4

PATIENT	SIZE AND POSITION OF CHROMOSOME 15 DELETION	ADDITIONAL CHROMOSOME ABERRATION	SEX	CLEFT PALATE	HEART DEFECT	DEVELOPMENTAL DELAY	FACIES ANOMALIES	ADDITIONAL ABNORMALITIES
**Present case**	del(15)(q14) **(submicroscopic, 1,6 Mb)**	t(1;13)(p31.1;q13),t(3;19)(p23;p12), t(9;15)(p23;q14),t(14;18)(q22;p11.23)	M	+	-	+	+	-

**Chen et al., 2008**	del(15)(q14) (submicroscopic, 5,6 Mb)	-	M	+	VSD	+	+	Epilepsy; speech and language disorder

**Brunetti-Pierri et al., 2008 case 1**	del(15)(q14) (submicroscopic, 4,2 Mb)	-	M	+ (Bifid uvula)	+	+	+	Bilateral inguinal hernias, autistic spectrum behavior

**Brunetti-Pierri et al., 2008 case 2**	del(15)(q13-q14) (submicroscopic, 8,9 Mb)	-	F	-	+	+	+	Hypotonia,feeding difficulties

**Erdogan et al., 2007**	del(15)(q14) (submicroscopic, 5,3 Mb)	-	F	+	ASD	+	+	Low-set ears, OFC 3rd percentile

**Galan et al., 1991**	del(15)(q12-q14)	-	M	+ (Bifid uvula)	Pulmonary valve stenosis	+	+	Right cryptorchidism, hearing deficency

**Tonk et al., 1995**	del(15)(q12-q14)	-	M	+	VSD, PDA, ischemic cardiomyopathy	+	+	Large fontanelles, hearing deficency

**Autio et al., 1988**	del(15)(q13-q15)	-	M	+	ASD	+	+	Cryptorchidism, kidney defect, corpus callosum agenesis

**Herva and Vuorinen, 1980**	del(15)(q12-q14)	Mosaic with 46, XY	M	-	VSD, hypoplastic pulmonary artery, atretic tricuspid valve	Died at 7 days	+	Cryptorchidism

**Pauli et al., 1983**	del(15)(pter-q15)	del(11)(q25-qter)	M	+ (Bifid uvula)	VSD	+	+	Cryptorchidism, unilateral renal ptosis

**Windpassinger et al., 2003**	del(15)(pter-q14)	del(3)(qter)	M	-	Persistent foramen ovale, PDA	+	+	Cryptorchidism, clubfeet, strabismus

**Kucerova et al., 1979**	del(15)(pter-q15)	del(3)(pter-p25)	M	-	-	+	+	Cryptorchidism

**Duckett and Roberts, 1981**	del(15)(pter-q14or15)	Trisomy 13(pter-q32 or 33)	F	+ (Bifid uvula)	VSD, ASD, PDA, transposition of great vessels	Died at 14 hr	+	Microphthalmia, tracheo-oesophageal fistula

**Ming et al., 1977**	del(15)(pter-q21)	del(6)(q27-qter)	F	+	-	Died at 3 days	+	Microphthalmia

**Schwartz et al., 1985**	del(15)(pter-q14)	del(22)(pter-q13.2)	M	-	Coarctation of aorta, PDA	+	+	Cleft alveolar ridge, hydronephrosis

**Matsumura et al., 2003**	del(15)(pter-q14)	Trisomy 22q	M	-	PDA	+	+	Renal failure, cryptorchidism

Legend: M: male; F: female; VSD: ventricular septal defect; ASD: atrial septal defect; PDA: patent ductus arteriosus; OFC: occipito-frontal headcircumference.

**Figure 5:**
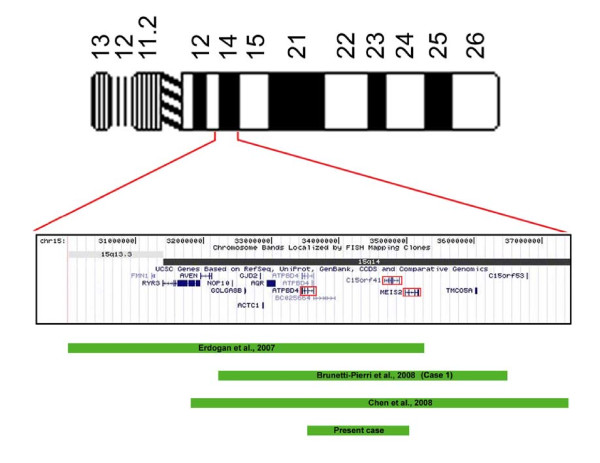
**Zoom in 15q14**. Map of the investigated region (red intervals starting from chromosome bands 15q13.3 and 15q14; from the UCSC database). Green solid bars represented the extent of the deletions observed in the cases reported in the literature. The genes included in the deleted region of our patient are surrounded by red frames.

Although our patient has a chromosomal complex karyotype, his phenotypic features largely overlap with patients having the 15q14 microdeletion as the sole chromosomal abnormality, suggesting a minor role of CCR on clinical presentation. To our knowledge three patients with the 15q14 interstitial deletions as their unique cytogenetic abnormality, have been previously described (Figure 5). Erdogan [37] reported a girl with moderate mental retardation, heart defect, cleft palate, minor facial dysmorphisms and developmental delay associated with interstitial microdeletion of 5.3 Mb. Brunetti-Pierri [3] described a child (case 1) with mild dysmorphic features, cleft palate/bifid uvula, congenital heart defects, psychomotor developmental delay with a microdeletion of 4.2 Mb. Chen [38] described a boy with speech and language disorder, facial dysmorphisms, cleft palate, epilepsy, ventricular septal defect, mental retardation and developmental delay with a microdeletion of 5.6 Mb. The microdeletion we have found is the smallest since it is 1.6 Mb in length.

Our patient exhibits only some clinical features in common with the cases with partially overlapping deletions, particularly mental retardation, speech defect and cleft palate (Figure 5). Specific facial anomalies in patients with 15q14 microdeletions include bitemporal narrowing, smooth philtrum, pointed chin and dysmorphic ears. Short stature is a characteristic feature. The present patient has normal birth parameters, whereas most previous reports have intrauterine growth retardation. Congenital heart defects or epilepsy are absent being probably related to patients with larger deletions. Thus, excluding epilepsy, which was reported only in Chen's paper, the unique clinical difference between the present case and the other three (Erdogan, Brunetti-Pierri -patient 1- and Chen) is the absence of cardiac malformations.

Morover, all the patients displayed cleft palate, suggesting that the deletion of 15q14 is correlated with this defect.

The mechanisms underlying CCRs formation are still poorly understood; some studies propose models based upon the principle of parsimony and the minimum amount of breaks required for the formation of the CCRs. Moreover, the proximal region of the long arm of chromosome 15 has a complex organization and undergoes recurrent nonhomologous recombination events that are facilitated by large repeat units, known as duplicons [39]. Another mechanism recently described as an alternative cause of genomic disorders with non-recurring breakpoints is Fork Stalling and Template Switching (FoSTeS), that initiates by a single strand DNA replication error in contrast to the meiotic recombination mechanisms [40]. Other mechanisms have been proposed to explain CCRs and they have been summarized by Zhang et al [13]. However, because the breakpoint sequences of CCRs have not yet been experimentally determined, the relationship between genomic architecture and the formation of the CCRs, along with the ability to infer the underlying mechanisms producing the rearrangements, remains elusive.

Interstitial deletion of chromosome 15 encompassing q14 is rare. Comparing the characteristics of our patient with those of cases currently reported in the literature, we have identified the smallest genomic region of overlap and we have recognized the related common phenotypic features. Both these observations suggest that this genetic lesion could reveal a novel recurrent syndrome.

## Consent

Written informed consent was obtained from the patient's relatives for pubblication of this case report and any accompanying images. A copy of the written consent is available for review by the Editor-in-Chief of this journal.

## List of Abbreviations

CCR: complex chromosome rearrangement; array-CGH: array-based comparative genomic hybridization; FISH: fluorescence in situ hybridization; BAC: bacterial artificial chromosome; STR: short tandem repeats; CNVs: copy number variations.

## Competing interests

The authors declare that they have no competing interests.

## Authors' contributions

MCR, MCD and AA made substantial contributions to conception and design, acquisition, analysis and interpretation of the data. MCD and RC performed the clinical evaluation. MCR, CS, MCD and AA drafted the manuscript. MCR, CS, AL and PS carried out the cytogenetic studies. CS, GDE, ACT and SP performed molecular analysis. All authors read and approved the final manuscript.
